# Uncharted waters: mesenchymal stem cell treatment for pediatric refractory rheumatic diseases; a single center case series

**DOI:** 10.1186/s12969-021-00575-5

**Published:** 2021-06-10

**Authors:** Stephen C. Wong, Leah C. Medrano, Alice D. Hoftman, Olcay Y. Jones, Deborah K. McCurdy

**Affiliations:** 1 Division of Allergy, Immunology, and Rheumatology, Department of Pediatrics, University of California Los Angeles, Los Angeles, CA 90095 USA; 2 Division of Rheumatology, Department of Pediatrics, University of Washington/Seattle Children’s Hospital, Seattle, 98105 USA; 3 Department of Pediatrics, University of California Los Angeles, Los Angeles, CA 90095 USA; 4 Division Pediatric Rheumatology, Department of Pediatrics, Walter Reed National Military Medical Center, Bethesda, MD 20889 USA

**Keywords:** Mesenchymal, Stem cell, Lupus, Arthritis, Treatment, Novel, Pediatric, Refractory

## Abstract

**Background:**

With the advent of innovative therapies including biologics and Janus kinase inhibitors, children with rheumatic diseases are more likely to have improved outcomes. Despite these advances, some children do not respond, or they, or their parents fear adverse events and seek other alternatives. Increasingly, private companies are offering mesenchymal stem cells (MSC) as an alternative, which are described as natural therapies for rheumatic diseases, often insinuating them as a cure. MSC have immunomodulatory properties, and transplantation of these stem cells have been used to successfully treat immunologic conditions like graft-versus-host disease. Lately, MSC research in adult lupus has been encouraging, but the clinical trials are still underway and in most, MSC therapy is not a standalone treatment. This retrospective case series will highlight three cases of pediatric refractory autoimmune disease whose parents sought out and received MSC therapy as a self-decision without first seeking medical advice from our specialty. The three families felt that their children were improved and in two believed that their child was cured. MSC have the potential of beneficial immunomodulation and may be a powerful tool in the therapy of rheumatic disease, but well controlled clinical trials are necessary and should be designed and monitored by experts in childhood rheumatic disease.

**Case presentation:**

Three children with three different rheumatic diseases; systemic lupus erythematosus, mixed connective tissue disease and juvenile idiopathic arthritis were under the care of pediatric rheumatology at a large, tertiary-care, teaching institution. Multiple non-biologic and biologic disease-modifying anti-rheumatic drugs failed to significantly decrease disease activity, and as a result, the families chose to undergo MSC therapy. After transplantation, all children improved per patient and parent report and tapered off conventional immunosuppressive drugs. No serious adverse events occurred in these three patients.

**Conclusion:**

The three cases presented in this report reflect comparable beneficial outcomes and minimal risks published in adult studies. These were not controlled studies, however, and benefit was reported rather than documented. These cases suggest that MSC transplantation may prove a promising adjunctive treatment option; however, further research, development of standardized infusion therapy protocols, and well-designed monitored clinical trials are essential.

## Introduction

The treatment of pediatric rheumatic diseases has advanced greatly over past two decades with the discovery and widespread use of biologics, allowing improved disease control in many cases. However, a significant number of patients, including those with systemic lupus erythematosus (SLE), mixed connective tissue disease (MCTD) and juvenile idiopathic arthritis (JIA) continue to have significant disease activity despite maximized usage of conventional therapeutic agents. Further investigations are needed for exploring new treatment modalities to improve patient outcomes.

Transplantation of mesenchymal stem cell (MSC), also known as medicinal signaling cells, has been used to treat steroid-refractory graft-versus-host disease (GVHD), while studied for its potential efficacy in many different illnesses ranging from multiple sclerosis to myocardial infarction with promising results [1]. MSC are known to have immunomodulatory properties and theoretically may ameliorate rheumatic diseases by suppressing T cell proliferation, B cell activation, and inflammatory cytokine production [2–4]. It is equally important to know, however, that while beneficial immunomodulation is most often noted, under certain conditions and in a different milieu, MSC may be pro-inflammatory [5]. With the anti-inflammatory and immunosuppressive properties in mind, researchers have investigated how MSC treatment would affect the course of SLE in certain adult populations with promising results [6–8]. Furthermore, a recent systematic review of MSC treatment in refractory adult rheumatoid arthritis ultimately only uncovered 4 relevant studies, which ultimately suggested MSC had a short-term benefit in treating rheumatoid arthritis [9]. Since MSC research remains a political, cultural, and religious area of contention, the breadth of research, especially in the pediatric population, is scarce within the United States. In this retrospective case series, we present our experience of MSC treatment in three patients who independently undertook this treatment after second opinion visits to commercial clinics licensed in the US or abroad.

## Case presentation

### Case 1

A previously healthy 9-year-old female presented with right eye pain and redness, and was diagnosed with idiopathic scleritis by ophthalmology and required steroid eye drops intermittently for flares. She was referred to pediatric rheumatology and her exam was normal, except for the scleritis. Her initial laboratory evaluation showed a positive antinuclear antibody (ANA) of 1:160 speckled, but all other labs were negative. She had a history of SLE in a maternal cousin, but no other autoimmune disease in the family. Three years into her illness, she developed polymyositis with severe arthritis with elevated muscle enzymes, speckled ANA of 1:640, and negative lupus serologies for double stranded (ds) DNA and extractable nuclear antigen (ENA). She was diagnosed with undifferentiated connective tissue disease (UCTD) and started on methotrexate and meloxicam with minimal efficacy.

Over the next 9 months, she progressed to develop a malar rash, a discoid rash and sustained leukopenia and lymphopenia. The discoid rash was biopsied and showed histologic features suggestive of lupus dermatitis. Her diagnosis was subsequently refined to SLE, meeting American College of Rheumatology 1997 revised criteria with malar rash, discoid rash, arthritis, positive ANA, and hematologic abnormalities. Her treatment included hydroxychloroquine and prednisone followed by disease-modifying anti-rheumatic drugs (DMARDs) and biologics as steroid-sparing medications including methotrexate, leflunomide, abatacept, tocilizumab and azathioprine over the following 48 months with limited efficacy. She continued to have recurrent scleritis and active arthritis causing significant pain. Her SLEDAI-2 K score was 6 and she had recurrent scleritis.

Her parents, exploring the internet to find a cure, decided to seek stem cell transplantation and found a company based in the United States where the cells are prepared, but given intravenously to the patient in Mexico. The patient was taken to a center near her home where she had liposuction to harvest adipose tissue. From the adipose tissue, the MSC were isolated and prepared for an autologous adipose tissue-derived MSC transplantation. She received one intranasal injection (100 × 10^6^ cells; approximately 1.8 × 10^6^ cells/kg/dose), one lymph node injection (100 × 10^6^ cells, approximately 1.8 × 10^6^ cells/kg/dose) three intravenous transfusions (379 × 10^6^, 256 × 10^6^, and 393 × 10^6^ cells) given within 1 week, then one further intravenous transfusion (234 × 10^6^ cells) 9 months later. While she was on MSC treatment, all conventional immunosuppressive medications were discontinued. After receiving the first MSC transplantation, she had a transient flare with arthralgia, malar rash, and recurrence of scleritis, which subsequently resolved in 2 months without treatment. One month after her first transplant, she had arthralgias, but no arthritis and her SLEDAI-2 K score was 0. After the second MSC transplant, she had transient scleritis, but did well and 1 month after, her SLEDAI-2 K score was 0. Otherwise, she had no other minor or serious adverse effect from MSC transplantation. At 17 years old, she is doing well and denies photosensitivity, rashes, stiffness, or joint pain. At 2 year follow-up after MSC, she has remained clinically stable; patient global assessment (PGA) decreased from 8/10 to 1/10 after treatment and physician global assessment from 7/10 to 2/10. The ANA titer declined from 1:640 to 1:80–160. Currently, she is only on intermittent topical ocular prednisolone therapy for rare patient-reported scleritis episodes and continues to have regular visits to pediatric rheumatology and ophthalmology clinics for routine monitoring.

### Case 2

A 19-year-old female was diagnosed with MCTD consisting of SLE and systemic sclerosis features at 15 years old. Initial presentation included intermittent fevers, Raynaud’s phenomenon, cervical lymphadenopathy, arthritis in her metacarpophalangeal joints, proximal interphalangeal joints, wrist, hips, and knees with generalized fatigue, weakness, dyspnea, and photosensitivity. Laboratory evaluation revealed high titer ANA (> 1:1280, Speckled), positive serologies for dsDNA and ribonucleoprotein antibodies and positive rheumatoid factor (RF). Ultrasound-guided lymph node biopsy showed benign reactive lymphadenopathy; no malignant cells or infectious organisms were identified. She was treated with multiple DMARDs and biologics including methotrexate, hydroxychloroquine, leflunomide, mycophenolate mofetil, rituximab, abatacept, and belimumab. She remained on intermittent, daily low to moderate dose oral prednisone throughout this time. Her response to treatment was marginal; her annual pulmonary function studies showed gradual reduction in diffusing capacity for carbon monoxide (DLCO) down to 41.5% predicted, although her high-resolution chest computed tomography and repeat echocardiograms remained normal.

**Fig. 1:**
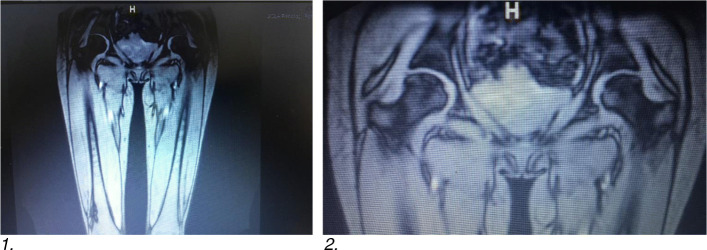
**a** MRI of bilateral lower extremities without contrast showing myositis of the quadriceps muscles. **b** Avascular necrosis of the femoral heads

Three years into her treatment, she developed significant pain and weakness. She was admitted and treated for polymyositis. She had been sedentary due to pain and weakness and had lost weight; her BMI was 18.6. She had diffuse myalgias and it was difficult to assess the muscle strength fully. The creatine kinase (CK) was slightly elevated at 285 U/L (range 38–282 U/L), the aldolase was 10 U/L (range 2.3–10.3 U/L), and the lactose dehydrogenase was high at 292 U/L (range < 24.1 U/L). Magnetic resonance imaging (MRI) revealed increased muscular edema within the gluteal, hamstring, quadriceps including the rectus femoris muscles. Also, of note, there was avascular necrosis of both hips without collapse of the femoral heads (Fig. 1). She was started on tocilizumab and received four doses administered intravenously every 14 days with improvement, but still with inability to walk, perform ADLs, or attend school. She was also given one dose of pamidronate that was well tolerated but did not decrease the pain in her hips. She was seen by orthopedics and plans were made for bilateral hip replacements. There was no noted skin thickening at that time and, although she did not have lupus and had no renal involvement, a SLEDAI-2 K was done with a score of 9, positive for myositis, arthritis, and leucopenia.

Due to poor quality of life from hip pain, she pursued MSC treatment. The MSC were derived from Wharton’s jelly after a cesarean birth and processed under a strict procedure to ensure viability, non-effectivity, and lack of genetic mutations. She received MSCs via intravenous transfusion as well as intraosseous injections directly into the head of the femurs bilaterally. Three hip injections (1 cc of Wharton’s Jelly MSC; 1.1 × 10^6^ MSCs) were given at months 0, 2, and 6, while intravenous transfusions (60 × 10^6^ MSCs; 1.2 × 10^6^ MSCs/kg/dose) were given at months 2 and 6. She continued her twice monthly intravenous tocilizumab during the first 2 months of MSC treatment; all conventional immunomodulatory therapy was discontinued thereafter since the patient stated that tocilizumab was making her wrists more swollen.

The patient reported fatigue and local pain at the site of the hip injections for 3 days but did not have any minor or serious adverse effect. Patient reported an improvement with a pre-treatment PGA of 7/10 and post-treatment PGA of 2/10. One month post the MSC therapy, she had resolution of the myositis and arthritis, but still had mild leucopenia, with a SLEDAI-2 K score of 1. Her ANA and RNP remained high titer positive. She had previously dropped out of her college semester pre-transplant and was able to return to full time studies and dance. Although her serologies remained unchanged, the erythrocyte sedimentation rate (ESR) normalized for 3 months after initial MSC therapy and remained only mildly elevated compared to pre-treatment levels. Although there continues to be radiographic evidence of avascular necrosis of the hips, there has been no collapse of the femoral head and the scheduled bilateral hip replacement surgery has been cancelled. Of concern, 2 years post-transplant, patient and family refuse conventional medicine, but she has signs of disease activity, now with skin thickening and a Rodnan skin score of 10.5. The family is planning, but currently cannot afford another MSC transplant.

### Case 3

An 18-year-old female with diagnosis of RF, cyclic citrullinated peptide antibody, and HLA-B27 positive JIA presented initially at 11 years of age with right knee arthritis and progressed to arthritis in bilateral knees, left elbow, and cervical spine. Her arthritis correlated with increased ESR. Her treatment included non-steroidal anti-inflammatory drugs (naproxen, sulindac, meloxicam), followed by methotrexate (initially oral, then subcutaneous). She transferred her care to another institution for 2 years, where she began etanercept with methotrexate. She had a brief remission 3 years into her initial diagnosis. A routine MRI of her elbow then showed active synovitis and new erosions, with recommendation for escalation to another biologic therapy (adalimumab). However, she had severe localized pain with injections, prompting the family to explore stem cell therapy abroad. She transferred back to our institution due to disagreement about therapies and parental concern about the high PGA of 6/10.

Despite counseling against untested therapies abroad by the rheumatology team, the family found a regenerative medicine clinic outside of the US and she underwent an umbilical cord MSC transplant at the age of 16 years old. The transplant involved three intravenous infusions over 4 days with cells derived from donor human umbilical cord tissue-derived MSC (120 × 10^6^ MSCs; 1.8 × 10^6^ MSCs/kg/dose). The transplant physician instructed the patient to wean off her rheumatic medications prior to the transfusion. She had no initial side effects, though developed a migratory arthritis flare 5 weeks post-transplantation which was attributed to a Herxheimer reaction by the transplant physician. No other minor or serious adverse effects were reported.

An MRI of her left elbow 5 months post-transplantation revealed continued progression of erosive changes. Post-transplantation labs remained unchanged, but there was a normalization of her ESR. One year after her initial transplant she again traveled for a second MSC intravenous transfusion (120.6 × 10^6^ cells; 1.9 × 10^6^ MSCs/kg/dose) due to continued elbow arthritis. She developed flu-like symptoms with this treatment. Follow-up 3 months later showed worsening elbow contracture and arthritis on MRI, though no worsening of erosions. She had an intra-articular steroid injection to the elbow joint the following month with marked improvement in her symptoms. Family decided to stop all conventional immunosuppressive therapy and initiate a complement of holistic management including monthly hyperbaric oxygen and red-light therapy with in-home lymphatic draining twice weekly and daily pulsed electromagnetic field therapy. Her most recent PGA is 1/10 and the family is content with her current condition.

*Summary Table:*

**Table:** 

	PATIENT ID*		
	***1***	***2***	***3***
***DIAGNOSIS***	*SLE*	*MCTD*	*JIA*
AGE AT** (years)			
***ONSET***	*9*	*15*	*11*
***MSC TX***	*14*	*17*	*16*
***FOLLOW UP***	*16*	*19*	*18*
***MSC TREATMENT***			
***SOURCE***	*Autologous Adipose tissue*	*Allogeneic Wharton’s Jelly*	*Allogeneic Umbilical Cord*
***DOSE***	*3.3 × 10*^*6*^*/kg/dose*	*1.2 × 10*^*6*^*/kg/dose*	*1.8 × 10*^*6*^*/kg/dose*
***ROUTE***	*IV ×3, intranasal ×1, lymph node ×1*	*IV ×2, IA × 3*	*IV ×4*
***PGA***			
***PRE TX***	*8/10*	*10/10*	*6/10*
***POST TX***	*2/10*	*2/10*	*1/10*
***DISEASE ACTIVITY***			
***PRE TX***	*SLEDAI-2 K 6*	*SLEDAI-2 K 9*	*JDAS 11*
***POST TX***	*SLEDAI-2 K 0*	*SLEDAI-2 K 1*	*JDAS 6*

* ID, identification number; Tx, treatment; IA, intraarticular; **Age in years

## Discussion and conclusions

As patients and parents advocate for themselves, physicians must become aware of therapies offered that are unconventional and unregulated. Some of these therapies may prove beneficial when studied under carefully regulated clinical trials, but some may prove harmful. The patients presented in this series had years of poorly controlled disease and failed multiple non-biological and biological DMARDs prior to initiating MSC treatment. In each case, the parents or patient became frustrated with conventional therapies and sought out therapies in this country or others that promised a cure. The clinics they found were quite costly, but all boasted of improved quality of life without or with much less disease and pain. Fortunately, in this series, all the MSC transplants were well tolerated and no patient had serious adverse effects such as infection, emboli, ectopic growth, or malignancy. Although some mild adverse effects were endorsed, all patients felt that MSC treatment was beneficial in achieving disease remission as noted with drastic improvement in patient global assessment. It should be remarked that disease remission may be confounded by conjunctive treatment as seen in case 2 (co-administration of MSC and a short trial of tocilizumab) and case 3 (an intra-articular steroid injection after MSC treatment). In addition to clinical improvement, laboratory changes revealed normalization of ESR, although specific autoantibody titers did not change.

There are many similarities that become apparent when comparing these three patients who received MSCs. Although each patient had unrelated pediatric rheumatic diseases, the perceived benign potential benefit of MSC and how they can modulate the immune response led families to choose this experimental and adjunctive treatment regimen for their children. The decisions to adopt MSC treatment was difficult since stem cell research is lacking in the field of pediatric rheumatology. A recent case series of 6 refractory JIA patients demonstrated MSC was safe, and though improvement was noted in multiple outcome variables, caution in interpreting benefit was mentioned as the study was non-blinded [10]. Macrophage activation syndrome was also seen in one of the patients with systemic-onset JIA. Another recent study also showed MSC led to reduction in inflammatory cytokines, improved immune network effects, adjusted immune tolerance, and alleviated symptoms in 10 children with JIA [11]. These two studies do not provide sufficient evidence for recommendation of MSC therapy in JIA or any rheumatic disease. Hence, none of these patients nor their families were recommended by their care team providers to pursue MSC therapy; the decision was solely made by the family, against medical advice. All families paid for MSC treatment out-of-pocket; current treatment is very expensive and averages in the tens of thousands of dollars per transfusion. One family sold their car, and a second family re-mortgaged their home to pay for the treatment they felt was necessary to alleviate their child’s rheumatic disease. In addition, when the disease became more active in patient 2, conventional therapies were refused with the plan to seek out another MSC treatment when the family can afford it.

MSCs are multipotent progenitor cells that can differentiate into various cell lines, including myocytes, adipocytes, chondrocytes, and osteoblasts under proper in vitro conditions. They were first hypothesized in the late nineteenth century but was formally discovered in the 1970s by Alexander Friedenstein [12, 13]. In 1991, Arnold Caplan, Ph.D., first named these cells MSCs [14]. Historically, MSCs have been isolated from cell cultures, but their native residence in vivo remained unknown for many years. It was not until the revelation of vascular pericytes (cells that enclose small blood vessels like capillaries) and their expressed MSC markers CD73, CD90 and CD105 that MSCs were discovered in vivo [15, 16]. MSCs are harvested from donors (allogeneic) or from the patient (autologous), mostly from bone marrow, adipose tissue, and placental/umbilical cord. Tissue banks and harvesting facilities have specific criteria and procedures for procurement of MSCs for clinical use, but no universal guidelines for the manufacturing process exist leading to heterogenous MSC yields [17].

MSC transplantation has remained a very specialized treatment modality with which few rheumatologists feel comfortable utilizing. Regulating authorities globally have investigated its potential for treatment in a whole host of diseases. Canada, New Zealand, and some European Union countries have approved Prochymal (Produced by Megaloblast and is an allogenic culture expanded adult MSC derived from bone marrow), while in Japan, Temcell (a similar product to Prochymal) has been approved for GVHD [18]. In the United States, MSC have been conferred orphan drug designation by the US Food and Drug Administration but has not been fully approved. Furthermore, no standard dosing has been published for pediatric rheumatic disease. There have been a few studies investigating the clinical efficacy and safety of MSC treatment in adult SLE with intravenous doses ranging from 1 × 10^6^ MSCs/kg/dose to 2 × 10^8^ MSCs/kg/dose [19]. In this case series, dosages ranged from 1.2 × 10^6^ MSCs/kg/dose to 6.5 × 10^6^ MSCs/kg/dose. In contrast, recommended dosing for MSC therapy in acute GVHD is 1 × 10^6^ MSCs/kg/dose to 2 × 10^6^ MSCs/kg/dose weekly for a total of 4 doses. Studies have shown that providers do not tightly adhere to recommended dosing of GVHD with an average dose of 1.4 × 10^6^ MSCs/kg and three total doses administered (range is 1–10 doses administered) [20]. With such varying doses in MSCs, adverse events might be expected in the comparatively higher-dosed infusion, but overall, studies have not shown significant adverse events, which is consistent with our observations [19, 20]. Yet, there are concerns that MSCs exhibit both a proinflammatory and an anti-inflammatory effect depending on conditions of culture and the donor’s immune milieu. While MSCs are thought to be primarily anti-inflammatory, there are studies that suggest that there are two subtypes of MSCs, one of which is pro-inflammatory, MSC1 [21]. It is reported that MSC1 induces a proinflammatory MSC phenotype that releases monocyte chemoattractant protein-1 (MCP-1), which increases the M1 proinflammatory phenotype. M1 macrophages secrete many cytokines, including interleukin-1β, tumor necrosis factor-α, and vascular endothelial growth factor, leading to an inflammatory state [21–26]. In addition, there has been some concern for tumorigenic potential of MSCs, but a recently published systematic review did not find this correlation to be significant; no serious adverse events were reported in this study [27]. Although the conditions required for optimal MSC therapy are not worked out, these factors must be considered in any clinical trial. Further research and controlled clinical trials with mandatory reporting of adverse events are essential and will assist pediatric rheumatologists in weighing the risks and benefits to this new treatment modality. Standardized procurement, preparation, cell viability, and the number of cell cycles and treatment protocols should be established for pediatric rheumatic disease. In addition, the necessity of stopping standard therapies must be studied as patients risk potentially life-threatening disease flares.

This case series demonstrates variability in the utilization of MSCs transplantation in the source of cells, types of cells, and route of administration. Two patients received allogeneic umbilical tissue-derived MSCs, while one patient received autologous adipose tissue-derived MSCs. Recent research has suggested that allogeneic MSC transplantation may be more immunosuppressive than autologous MSCs. MSCs derived from lupus patients have shown dysfunction in both proliferation and immunoregulation and appear to be phenotypically senescent [28]. All patients received systemic treatment via intravenous route, though two patients also received local injection (joint, lymph node, and intranasal). Local administration leads to increased complexity of differentiated engrafted MSCs due to microenvironmental milieu. On the other hand, systemic treatment allows infused MSCs to traffic to injured and inflamed tissues through chemokines and growth factors [29]. Dosing intervals also ranged from monthly to yearly infusions. Since there has been a scarcity of MSC research in pediatric rheumatology, variability is understandable. With an increase of MSC treatment publications in the past few years, more families will continue to seek this emerging therapy for their children with refractory disease, despite the experimental status of MSC transplant. This study aims to bring awareness, and caution, of this novel treatment to the pediatric rheumatology community as families will often seek this therapy without informing the treating team, stopping all medications prior to transplant without physician knowledge. In addition, the transplant physicians in these three cases did not communicate with the primary rheumatology team prior to stopping previous medications and initiating transplant. Although stem cell therapy remains controversial, especially in the United States, ongoing research and close scrutiny of the viability of MSC in ameliorating refractory pediatric rheumatic disease are necessary for the safety of all patients given the above considerations.

This retrospective series highlights the potential of mesenchymal stem cell therapy in refractory pediatric rheumatic diseases. The immunomodulatory effects of MSC treatment on autoimmune inflammatory disease make it a promising adjunctive treatment modality in certain cases. These three families took the chance and overcame multiple barriers (ie. expensive cost, travel out of the country, risk of disease flares, risk of side effects in uncontrolled settings) as a self-decision. We are compelled to share this experience as a possible sign of changing public perception and a silent request for awareness. Based on this, we suggest further evaluation of the safety and efficacy of MSC therapy is warranted for our patients within the premises of our subspecialty.

## Data Availability

Data sharing is not applicable to this article as no datasets were generated or analyzed during the current study. Any case details used in the current study are available from the corresponding author on reasonable request.

## Abbreviations

SLE: Systemic lupus erythematosus
MCTD: Mixed connective tissue disease
JIA: Juvenile idiopathic arthritis
MSC: Mesenchymal stem cell
GVHD: Graft-versus-host disease
ANA: Antinuclear antibody
dsDNA: Double stranded deoxyribonucleic acid
ENA: Extractable nuclear antigen
UCTD: Undifferentiated connective tissue disease
DMARDs: Disease-modifying anti-rheumatic drugs
PGA: Patient global assessment
RF: Rheumatoid factor
MRI: Magnetic resonance imaging
ESR: Erythrocyte sedimentation rate
HLA: Human leukocyte antigen
